# CTLA4Ig Improves Murine iTreg Induction via TGF*β* and Suppressor Function *In Vitro*

**DOI:** 10.1155/2018/2484825

**Published:** 2018-07-02

**Authors:** Nina Pilat, Benedikt Mahr, Martina Gattringer, Ulrike Baranyi, Thomas Wekerle

**Affiliations:** Section of Transplantation Immunology, Department of Surgery, Medical University of Vienna, Vienna, Austria

## Abstract

Blockade of the CD28:CD80/86 costimulatory pathway has been shown to be potent in blocking T cell activation *in vitro* and *in vivo*. The costimulation blocker CTLA4Ig has been approved for the treatment of autoimmune diseases and transplant rejection. The therapeutic application of regulatory T cells (Tregs) has recently gained much attention for its potential of improving allograft survival. However, neither costimulation blockade with CTLA4Ig nor Treg therapy induces robust tolerance on its own. Combining CTLA4Ig with Treg therapy would be an attractive approach for minimizing immunosuppression or for possibly achieving tolerance. However, since the CD28 pathway is more complex than initially thought, the question arose whether blocking CD80/86 would inadvertently impact immunological tolerance by interfering with Treg generation and function. We therefore wanted to investigate the compatibility of CTLA4Ig with regulatory T cells by evaluating direct effects of CTLA4Ig on murine Treg generation and function *in vitro*. For generation of polyclonal-induced Tregs, we utilized an APC-free *in vitro* system and added titrated doses of CTLA4Ig at different time points. Phenotypical characterization by flow cytometry and functional characterization in suppressor assays did not reveal negative effects by CTLA4Ig. The costimulation blocker CTLA4Ig does not impair but rather improves murine iTreg generation and suppressor function *in vitro*.

## 1. Introduction

In order to exert a proper T cell immune response, the T cell needs at least two signals, namely, an antigen-specific signal via the T cell receptor (TCR) and a costimulatory signal provided by a number of specialized cell surface receptors [1]. One of the best studied costimulatory pathways is the CD28:B7 pathway, mediated by the binding of CD28, which is expressed on T cells, to B7 molecules (CD80 and CD86), expressed on antigen-presenting cells (APCs). Costimulation via CD28 induces proliferation, survival, and cytokine production, whereas lack of CD28 signaling following TCR ligation induces classical T cell anergy [2]. Physiologically, T cell activation leads to the upregulation of the negative costimulatory molecule cytotoxic T-lymphocyte-associated protein 4 (CTLA4), which binds B7 molecules with higher affinity and avidity, thereby providing a negative feedback mechanism, which prevents further CD28 signaling [3]. Additionally, CTLA4 is constitutively expressed on regulatory T cells (Tregs) being critical for suppressor function [4] and overall immune homeostasis [5]. As direct CD28 blockade is difficult to achieve, the fusion protein CTLA4 immunoglobulin (CTLA4Ig) was developed as an alternative strategy to indirectly block CD28 ligation [6], at that time still unaware of the importance of CTLA4 signaling. In light of the protolerogeneic functions of CTLA4, the therapeutic use of CTLA4Ig in tolerance protocols was put into question [7], as it prevents not only ligation of CD28 but also ligation of CTLA4, which is critical for Treg function [4].

CD4^+^CD25^+^FoxP3^+^ regulatory T cells (Tregs) are critical mediators of self-tolerance [8] and have been shown to prevent autoimmunity and to induce (transplantation) tolerance in numerous experimental animal models [9–11]. Therefore, Tregs would be promising candidates for the intentional induction of transplantation tolerance or as part of calcineurin inhibitor- (CNI-) sparing immunosuppressive regimens, as chronic use of CNIs—still being the backbone of current immunosuppressive regimens—is associated with substantial side effects, including profound nephrotoxicity. Furthermore, previous studies already demonstrated that CNIs inhibit Treg function and have markedly negative effects on Tregs [12], including decreased FoxP3 expression and demethylation status and subsequent impaired suppressive capacity [13].

Whereas CTLA4Ig has proven great potency in tolerance induction in various mouse models in combination with anti-CD40L [14], donor-specific transfusion (DST) [15], or bone marrow transplantation [16–18], it was less effective in nonhuman primate studies. However, its immunosuppressive efficacy, combined with the absence of renal toxicity, maximized its clinical relevance and enabled its successful use in clinical transplantation [19].

The combination of CTLA4Ig treatment and Treg cellular therapy seems an attractive approach for studies of minimization or even withdrawal of chronic immunosuppressive therapy. Several studies have investigated the effect of CTLA4Ig on Treg survival and potential effects on Treg function utilizing *in vitro* and *in vivo* models [20–22]; however, results are still inconclusive and conflictive. While most studies focused mainly on the effect on thymus-derived Tregs (tTregs), we used an in vitro model of transforming growth factor beta- (TGF-) induced Tregs (iTregs), which have been shown to be potent in the suppression of alloresponse in a mixed chimerism model for tolerance induction *in vivo* [17, 23]. In this study, we show that iTreg induction and suppressive potential of Tregs are not impaired by the presence of CTLA4Ig, therefore adding another piece of puzzle to the complex relationship between costimulation blockade of the CD28/CTLA4/B7 pathway and its effect on the different subsets of Tregs. Indeed, we provide evidence that the presence of CTLA4Ig rather enhances TGF*β*-mediated conversion towards a suppressive phenotype, indicated by expression of Treg-specific markers and suppressive function *in vitro*.

## 2. Materials and Methods

### 2.1. Animals

Female C57BL/6 (B6, H-2^b^) and BALB/C (H-2^d^) mice were purchased from Charles River Laboratories (Sulzfeld, Germany), housed under specific pathogen-free conditions, and used at 6 to 12 weeks of age. All experiments were approved by the local review board of the Medical University of Vienna and the Austrian Federal Ministry of Science, Research and Economy and were performed in accordance with the national and international guidelines of laboratory animal care.

### 2.2. Generation of Tregs

Tregs were generated as described previously [17]. Shortly, cells were isolated from spleen and lymph nodes of naïve B6 mice. For iTreg generation, CD4^+^ cells were isolated (L3T4 microbeads, Miltenyi Biotec) and cultured for 6 days (144 h) in precoated 24-well plates (100 *μ*g/ml anti-CD3 (145-2C11), 10 *μ*g/ml anti-CD28 (37.51); BD Pharmingen) in the presence of 100 U/ml IL-2 (Sigma) and 5 ng/ml rhTGF*β* (R&D Systems) [24]. Human CTLA4Ig (abatacept, purchased from Bristol-Myers-Squibb) was added at different concentrations (low dose, LD 40 *μ*g/ml; high dose, HD 200 *μ*g/ml) for the length of culture or for the last 24 h of culture (HD 200 *μ*g/ml). Due to intentionally introduced mutations to achieve higher avidity for human B7 molecules, belatacept lost effective binding capacity for murine B7; therefore, only abatacept is used in the current study [25]. Living cells were counted at indicated time points using CASY System (Innovatis). Purity of MACS-sorted populations was >90%. At the end of culture, the Treg-enriched cell populations were used for subsequent cell culture assays without additional sorting steps [17].

### 2.3. Antibodies and Flow Cytometric Analysis

Multicolor flow cytometric analysis of Tregs was performed as described previously [17]. Monoclonal antibodies (mAbs) with specificity against CD4 (RM4-4), CD25 (7D4), CD62L (Mel-14), and CTLA4 (UC10-4F10-11) were used. For intracellular staining, FoxP3 (FJK-16s) Staining Kit (eBioscience) was used according to the manufacturer's protocol. PI was used for dead cell exclusion when appropriate. Surface staining was performed according to standard procedures, and flow cytometric analysis was done on Coulter Cytomics FC500 using CXP software (Coulter, Austria) for acquisition and analysis.

### 2.4. *In Vitro* Suppression Assays

*In vitro* suppression assays were performed as described in detail previously [17, 26]. Briefly, 4 × 10^5^ responder splenocytes (B6) were cocultured in triplicates with decreasing numbers of iTregs (4 × 10^5^, 2 × 10^5^, and 8 × 10^4^ for a ratio of 1 : 1, 2 : 1, and 5 : 1 (responder cells versus Tregs)), in the presence of 4 × 10^5^ irradiated (30 Gy) allogeneic splenocytes (BALB/C). Alternatively, responder cells were stimulated polyclonally with anti-CD3 (clone 145-2C11 at 5 *μ*g/ml). Freshly isolated CD4^+^ cells cultured without recombinant human (rh) TGF*β* were used as control. After 72 h of incubation, cells were pulsed with [3H]-thymidine (Amersham, Biosciences, UK) for 18 h. Incorporated radioactivity was measured using scintillation fluid in a *β*-counter. Stimulation indices (SI) were calculated in relation to medium controls. Results represent averaged data of triplets from pooled animals.

### 2.5. T Cell Proliferation Assay

CD4 T cells were isolated from spleen and lymph nodes of B6 mice and enriched via magnetic bead-based positive selection (CD4 L3T4 microbeads, Miltenyi Biotec, Bergisch Gladbach, Germany). MACS-sorted cells had a purity > 95%. 4 × 105 CD4 T cells (B6) were cultured in triplicates in the presence or absence of CTLA4Ig (low dose, LD 40 *μ*g/ml; high dose, HD 200 *μ*g/ml) with or without high-dose IL-2 (1000 U, Sigma). Cells were polyclonally stimulated with anti-CD3 (clone 145-2C11 at 5 *μ*g/ml) for 72 h and pulsed with [3H]-thymidine (Amersham, Biosciences, UK) for 18 h as described for suppressor assays.

### 2.6. Cytokine Analysis

IL-10 and IL-17A were measured by enzyme-linked immunoabsorbent assays (ELISA). Supernatant of *in vitro* cultures was harvested at different time points and stored at −80°C until analysis. ELISA kits were used according to the manufacturer's protocol (eBioscience, San Diego, CA). Plates were measured at 450/595 nm using a VICTOR plate reader (PerkinElmer).

### 2.7. Statistics

A two-sided Student's *t*-test with unequal variances was used to compare results and SI values between groups. A *p* value less than 0.05 was considered to be statistically significant.

## 3. Results

### 3.1. CTLA4Ig Does Not Impair Proliferation of T Cells in the Presence of TGF*β*

**Figure 1:**
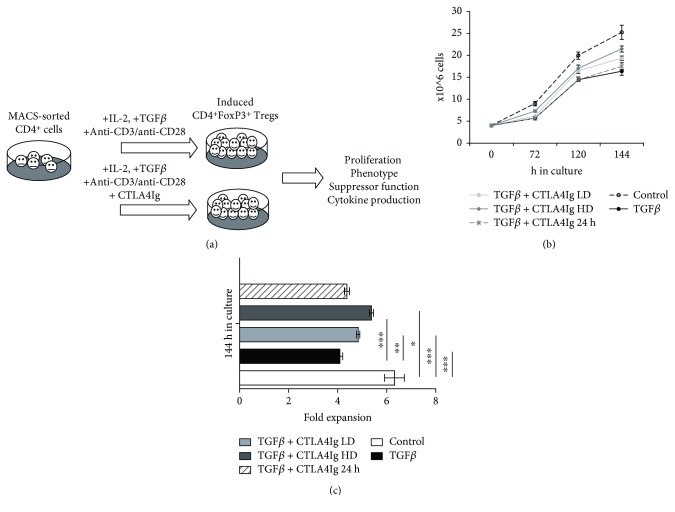
CTLA4Ig does not inhibit Treg proliferation *in vitro.* (a) Schematic illustration of Treg induction *in vitro* culture is shown. (b) Proliferation curve showing mean cell numbers for different culture conditions (all groups were stimulated with anti-CD3/CD28 in the presence of IL2) over time and (c) fold expansion after 144 h in culture are shown. Cells were plated in quadruplicates; control indicates CD4 T cells stimulated with anti-CD3/CD28 in the presence of IL2; results are representative for 3 independent experiments. Error bars represent standard deviation. ^∗^*p* < 0.05, ^∗∗^*p* < 0.01, and ^∗∗∗^*p* < 0.0001.

For addressing the specific question, whether CTLA4Ig interferes with Treg induction via TGFbeta (TGFb/TGF*β*), we used an *in vitro* model for the generation of induced Tregs (iTregs) that were previously shown to generate potent Treg populations which have been successfully used as cell therapy in a model of chimerism-induced transplantation tolerance [17, 23]. Moreover, it has been proposed that in vitro generation of iTregs via TGF*β* mimics the in vivo development of adaptive Tregs [27]. We added different amounts of CTLA4Ig to the Treg induction culture (schematic experimental approach outlined in Figure 1(a)), mimicking the therapeutic serum concentration observed in nonhuman primate renal transplantation (~30 *μ*g/ml serum levels → 40 *μ*g/ml chosen for low dose) [25]. These data also served as basis for the clinical studies leading to the approval of belatacept in human renal transplantation [19, 28, 29], strengthening the importance of this study for clinical translation. CTLA4Ig was added either at the beginning of *in vitro* Treg induction culture or 24 h before cells were harvested and used for further analysis. Net Proliferation of total CD4+ T cells was reduced when TGF*β* was added, which is consistent with previous findings. Importantly, CTLA4Ig had no detrimental effect on cell proliferation in the presence of TGF*β* (Figures 1(b) and 1(c)), whereas in the absence of TGF*β*, the same concentration of CTLA4Ig is sufficient to block T cell proliferation almost completely (data not shown and [14]). Moreover, we observed significantly increased proliferation in the presence of CTLA4Ig in a dose-dependent manner.

### 3.2. Induction of Regulatory Phenotype In Vitro Is Not Impaired by CTLA4Ig

**Figure 2:**
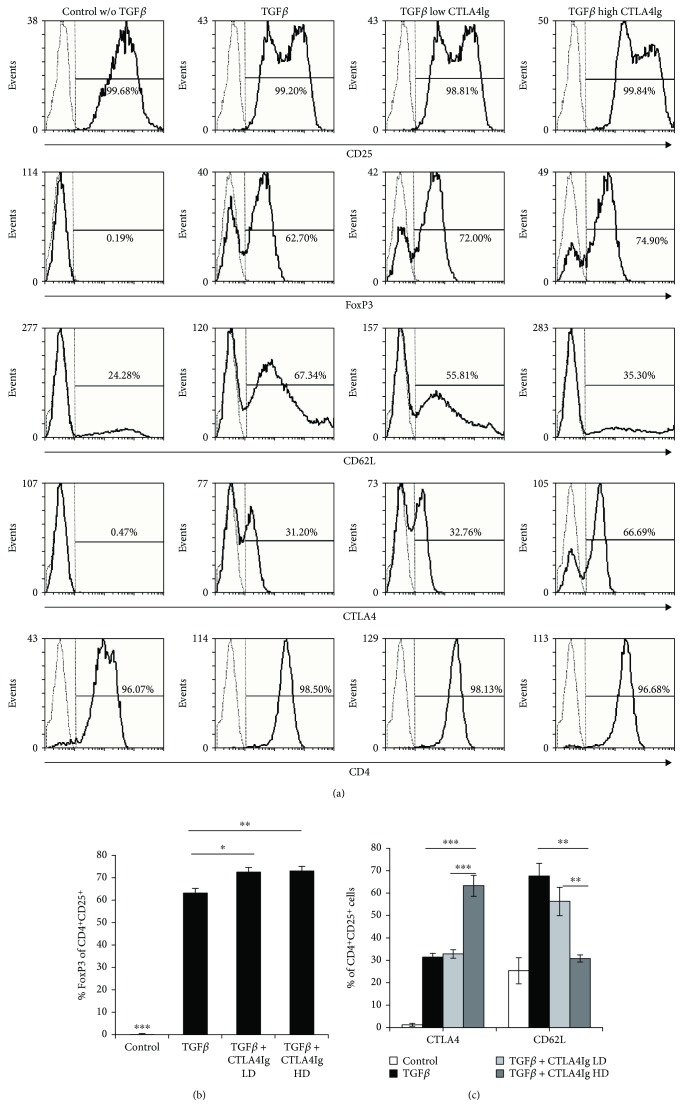
CTLA4Ig enhances the proportion of induced Tregs *in vitro.* (a) Representative histograms of Treg markers are shown for different culture conditions (gated on total leucocytes). CD4^+^CD25^+^ T cells were analyzed (b) for the expression of FoxP3 (indicating induction of regulatory phenotype) by intracellular FACS staining after 6 days of in vitro culture ± CTLA4Ig and (c) Treg-associated markers CTLA4 and CD62L, which were analyzed and compared between groups. Cells were plated in triplicates for each culture condition. Data are representative for 3 independent experiments; control indicates CD4 T cells stimulated with anti-CD3/CD28 in the presence of IL2. Error bars represent standard deviation. ^∗^*p* < 0.05, ^∗∗^*p* < 0.01, and ^∗∗∗^*p* < 0.0001.

Consistent with literature [24] and our previous results, TGF*β* induced a regulatory phenotype, indicated by de novo FoxP3 expression in the majority of CD4^+^ cells and upregulation of Treg-associated markers CD25, CD62L, and CTLA4 (Figures 2(a)–2(c)). The proportion of FoxP3-expressing cells, namely, CD4^+^CD25^+^FoxP3^+^ Tregs, was significantly higher in cultures containing TGF*β*, irrespective of the additional presence of CTLA4Ig (Figure 2(a)). Low-dose treatment with CTLA4Ig led to a significant increase in the percentage of CD4^+^CD25^+^FoxP3^+^ Tregs (Figure 2(b)), but there was no considerable effect on the expression of CD62L or CTLA4 (Figure 2(c)), which are both considered to be important for *in vivo* Treg function and are considered to be important surface markers of Tregs [4, 30]. High doses of CTLA4Ig on the other hand led to a significant increase in CTLA4 expression but also a significant decrease of CD62L expression (Figure 2(c)). Thus, the presence of CTLA4Ig does not impair but rather promotes induction of regulatory phenotype via TGF*β* and the expression of FoxP3.

### 3.3. Tregs Induced via TGF*β In Vitro* in the Presence of CTLA4Ig Are Not Impaired in Suppressor Function

**Figure 3:**
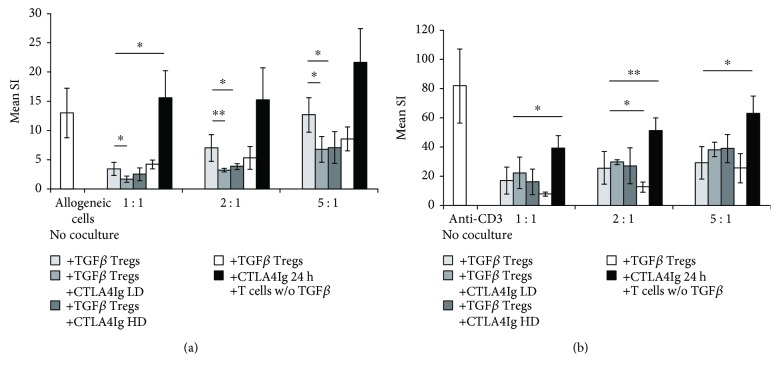
Suppressive potential of *in vitro* induced iTregs is not impaired by CTLA4Ig. For *in vitro* suppressor assays, titrated numbers of Treg-enriched cells (after cultivation with TGF*β* ± CTLA4Ig; Teff : Tregs) were added to 4 × 10^6^ naïve B6 responder cells (responder cells: Tregs). Responder cells were stimulated with (a) 4 × 10^6^ fully allogeneic BALB/C stimulator cells (irradiated) or (b) by polyclonal stimulation with plate-bound anti-CD3. Stimulation indices (SI; calculated in at least triplicates divided by pooled medium controls) of coculture suppression by Tregs induced in the presence of CTLA4Ig were compared to TGF*β* Treg controls. Results are representative for 3 independent experiments; error bars indicate standard deviation. ^∗^*p* < 0.05 and ^∗∗^*p* < 0.01 in comparison to TGF*β* Tregs w/o CTLA4Ig.

To test the suppressive potential of iTregs generated in the presence of CTLA4Ig, we performed coculture assays to determine their potential to suppress proliferation of naïve cells in response to allogeneic or polyclonal stimulation. Titrated numbers of in vitro induced iTregs ± CTLA4Ig were added to MLRs in which unseparated B6 responder splenocytes were stimulated with irradiated Balb/c cells (Figure 3(a)). We could show that iTregs induced in the presence of varying doses of CTLA4Ig suppressed T cell proliferation in response to alloantigen in a dose-dependent manner. In comparison to control iTregs, LD CTLA4Ig Tregs showed increased potential for suppression at all cell doses tested.

Next, we wanted to determine the potential of Treg to suppress polyclonal activation after T cell stimulation with anti-CD3. We could show that iTregs induced in the presence of CTLA4Ig were able to suppress T cell proliferation similar to iTreg controls (Figure 3(b)).

These findings imply that the presence of CTLA4Ig during Treg generation has no negative effect on the suppressor function of *in vitro* induced iTregs. Interestingly, there is a trend towards increased suppressor function by Tregs generated in the presence of CTLA4Ig in response to allogeneic rather than polyclonal stimulation.

### 3.4. CTLA4Ig Preserves the Ability to Produce IL-10 and Prevents Conversion to IL-17-Producing Cells

**Figure 4:**
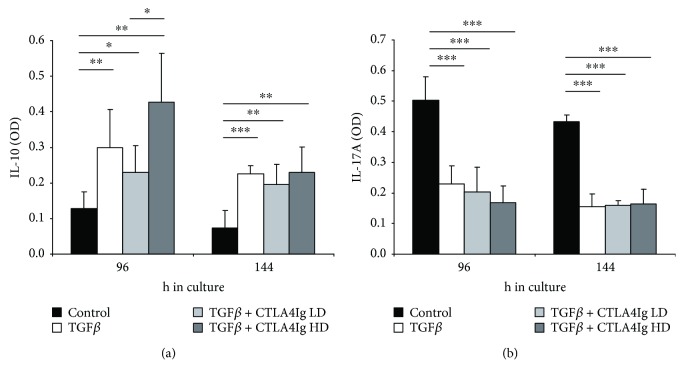
The presence of CTLA4Ig does not interfere with cytokine release *in vitro.* Supernatant of in vitro Treg induction cultures was collected at different time points and analyzed for the change in cytokine expression. (a) CD4^+^ T cell cultures cultivated in the presence of TGF*β* showed significant increase in the production of suppressive cytokine IL-10 and (b) a significant decrease in the production of inflammatory cytokine IL-17A. Data were obtained from cells cultivated in triplicates and are representative for 3 independent experiments; control indicates CD4 T cells stimulated with anti-CD3/CD28 in the presence of IL2. Mean values for optical density (OD) are shown; error bars indicate standard deviation. ^∗^*p* < 0.05, ^∗∗^*p* < 0.01, and ^∗∗∗^*p* < 0.0001.

Several reports have demonstrated that TGF*β*-induced iTregs can redifferentiate into FoxP3-negative conventional T cells upon restimulation in the absence of TGF*β*, which suppresses Th1 and Th2 differentiation [31]. Moreover, differentiation into IL-17-producing Th17 cells is not inhibited by the presence of TGF*β* and intermediate differentiation stage IL17^+^FoxP3^+^ T cells have been described [32]. We therefore aimed to determine whether the presence of CTLA4Ig affects the cytokine profile, especially the regulatory cytokine IL-10 and the inflammatory cytokine IL-17 (Figure 4(a) and 4(b)). The presence of CTLA4Ig during Treg generation did neither impair production of anti-inflammatory nor enhance production of proinflammatory cytokine IL-17, as determined by ELISA.

### 3.5. CTLA4Ig Suppresses T Cell Proliferation in the Absence of Antigen-Presenting Cells

**Figure 5:**
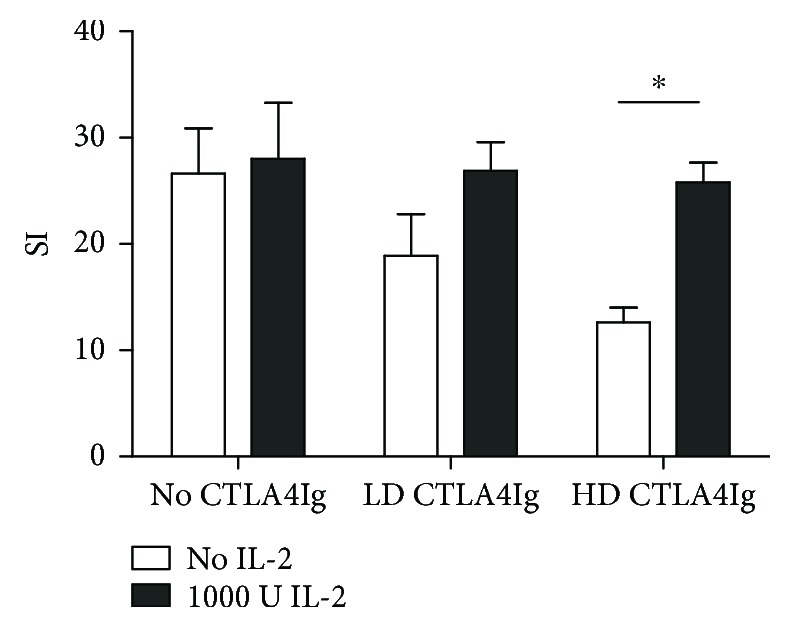
CTLA4Ig directly inhibits CD4 T cell proliferation *in vitro.* For proliferation assays, 4 × 10^6^ T naïve B6 responder cells were stimulated with plate-bound anti-CD3 in the presence or absence of high levels of IL-2. CTLA4Ig was added to T cell cultures at different doses. Stimulation indices (SI; calculated in at least triplicates divided by pooled medium controls) were compared to controls without costimulation blockade. Results are representative for 3 independent experiments; error bars indicate standard deviation. ^∗^*p* < 0.01.

The CTLA4Ig concentrations used in iTreg induction experiments have been previously shown to inhibit alloresponses *in vitro* by binding on B7 on APCs and therefore preventing T cell activation via CD28 [33]. However, little is known about the effect of CTLA4Ig on T cells as it is commonly assumed that B7 expression is restricted to APCs and activated T cells can also express B7 [34]. When we used CTLA4Ig in a polyclonal, APC-free proliferation assay, we revealed a dose-dependent inhibition of CD4 T cells; notably, this effect was impeded by high doses of IL-2 (Figure 5).

Taken together, these findings indicate that costimulation blocker CTLA4Ig does not negatively impact TGF*β*-mediated conversion of Tregs in terms of proliferation, FoxP3 expression, phenotype, *in vitro* suppressive capacity, or cytokine profile. In the experiments shown herein, we observed a positive effect on Treg conversion and suppressive capacity by the presence of CTLA4Ig, suggesting a possible interaction with B7 molecules expressed on T cells.

## 4. Discussion

Adaptive peripheral CD4^+^CD25^+^FoxP3^+^ Tregs (pTregs) can be deliberately generated from CD4^+^CD25 conventional T cells *in vivo* under conditions including the presence of suboptimal antigen concentration or antigen delivery via nonimmunogenic methods such as oral or intravenous injection, peptide pumps, or antibody-mediated DC targeting in the absence of adjuvants [35]. In this study, we tried to mimic pTreg generation under defined experimental conditions in an APC-free system in order to directly evaluate a possible impact of the costimulation blocker CTLA4Ig. Although there are substantial differences between *in vitro* induced iTregs and *in vivo* induced pTregs, we think that this study adds valuable mechanistic knowledge regarding a possible negative role of CTLA4Ig during Treg conversion.

The expression of B7 molecules is not only exclusively restricted to APCs but may also occur on T cells upon activation [34, 36]. The role of B7 on APCs has been thoroughly studied while their role for T cells remains largely unknown. Taylor et al. showed that B7 expression by T cells is essential for downregulating immune responses through CTLA4 [37]. In line with this, B7 knockout T cells are resistant to Treg-mediated suppression via the CTLA4 pathway [38]. Moreover, it has been reported that CTLA4Ig inhibits T cell proliferation in a purified CD4 T cell proliferation assay upon stimulation with anti-CD3 [39]. This observation suggests that CTLA4Ig either inhibits T-T cell interactions via the B7-CD28 pathway or induces a negative stimulus in the T cell. However, the short cytoplasmic tails of B7.1 and B7.2 question the latter assumption [40]. Considering that T cells do provide costimulatory help to each other, it seems conceivable that CTLA4Ig covers B7 molecules on T cells and thereby increases the available targets for the anti-CD28 antibody in the *in vitro* iTreg generation system. Costimulation via antibody cross-linking induces a supraphysiological signal which could hypothetically explain improved iTreg induction in the presence of CTLA4Ig [41].

Numerous reports have tried to uncover the relationship between Treg and CTLA4Ig after the introduction of the first rationally designed selective T cell costimulation blocker in the clinics. Initially designed for treatment of autoimmune diseases (abatacept; approved for rheumatoid arthritis in 2005), it was mutated to induce higher avidity binding—especially for CD86—for the prophylaxis of organ rejection (belatacept; approved for renal transplantation in 2011). Although CTLA4Ig was initially envisioned to induce tolerance towards solid organ allografts by selective T cell costimulation blockade, which was intended to lead to anergy and tolerance, concerns arose whether it has a potentially detrimental impact on Tregs. Recently, it has been shown that Tregs depend on CD28 signaling during development in the thymus [42]; however, this might be a concern for tTreg rather than pTreg development. Other data suggest that post maturational CD28 signaling is important for Treg function [43] which was demonstrated by the use of a Treg-specific CD28 conditional knockout mouse. Although these are vital data for the understanding of the CD28/CTLA4/B7 pathway, it does not exactly mimic the situation under CTLA4Ig treatment. Recently, it was postulated that CD28 signaling is the main driver behind Treg proliferation but CTLA4:CD80/CD86 interactions are also needed to control homeostatic proliferation [44].

Although the main function of CTLA4 *in vivo* is thought to be T cell extrinsic, there are multiple proposed mechanisms about additional cell intrinsic functions [40]. Uncovering of cell intrinsic functions is complicated by the fact that ligation by CTLA4-specific antibodies might not reflect physiologic balance of CTLA4/CD28 engagement with its natural ligands; nonetheless, several negative signaling pathways to intrinsically inhibit T cells proliferation have been identified. Although several negative signaling scenarios induced by anti-CTLA4 antibodies have been described, no cell intrinsic signals driven by natural ligands have been confirmed [45]; thus, it is rather unlikely that CTLA4Ig causes negative effects by inhibiting CTLA4 signaling.

Several studies intended to evaluate Tregs in transplant patients under belatacept treatment; however, concomitant immunosuppressive regimen complicates interpretation of these results. Whereas some groups reported no short- or long-term effects on Treg numbers and function when compared to treatment with CNIs [46, 47], others reported a decrease in Treg and FoxP3 mRNA levels [48]. The only conclusion from clinical experience with CD28 blockade via CTLA4Ig, which could be agreed on, was the fact that induction of tolerance with CTLA4Ig and current concomitant regimens was unlikely [13, 49]. In mouse models, on the other hand, CTLA4Ig treatment seems to be able to favor regulatory mechanisms in order to induce an operational tolerant state. When we examined the effect of costimulation blockade via CTLA4Ig on Tregs in a dose-dependent murine heart transplantation model, we found that although Treg numbers were initially decreased, they normalized under long-term treatment with CTLA4Ig and that there is a synergy between CTLA4Ig and Tregs when CTLA4Ig is given at nonsaturating doses [20]. Moreover, CTLA4Ig and Treg cell transfer act synergistically in an irradiation-free mixed chimerism model, which is strongly dependent on intragraft regulation [50]. *In vitro* studies have also shown immunomodulatory potency for CTLA4Ig by preservation of tTregs [22], promotion of Treg conversion [51], and inhibition of effector responses via a Treg/TGF*β*-dependent pathway [52]. On the other hand, some studies demonstrated that CTLA4Ig interferes with tolerance by the inhibition of Treg expansion [53, 54], suggesting that there is a complex relationship between CTLA4Ig treatment and Tregs and a better understanding is warranted before synergy between them can be predicted in a specific model. Another theory coming from autoimmune research, which is underlined by several reports, suggests that anergy (as induced by costimulation blockade) is an intermediate between auto-/alloreactive T cells that eventually become Tregs [55]. This is in line with the infectious tolerance model, which was proposed by Kendal and Waldmann [56].

Here, we have shown that CTLA4Ig does not negatively impact Treg conversion via TGF*β in vitro*, which in our opinion is of major relevance as it mimics the generation of allospecific pTregs in the periphery. Clinical data and murine studies suggest that in long-term kidney transplant patients, indirect allospecific T cells mainly contribute to late graft rejection [13, 57, 58]. As tTregs and pTregs are generally believed to represent distinct TCR repertoires, several reports have suggested a division of labor between those subsets [59]. It has been suggested that while tTregs mainly participate in the inhibition of T cell trafficking in the allograft, pTregs primarily prevent T cell priming by acting on APCs [60]. Our data clearly demonstrate that the presence of CTLA4Ig does not interfere with Treg conversion or proliferation *in vitro*. More importantly, Treg suppressive capacity as well as cytokine production is not impaired even with high doses of the costimulation blocker.

In summary, data from clinical trials using belatacept instead of CNIs show that both immunosuppressive regimens lead to a (transient) decrease of Tregs and impaired suppressor function. Nevertheless, impairment of Tregs is not worse under belatacept treatment, which results in better patient and graft survival [29, 61, 62], making it favorable over CNI-based immunosuppressive regimens.

## Data Availability

The data used to support the findings of this study are available from the corresponding author upon request.
